# Retraction: Structural equation modeling to estimate treatment adherence based on the light triad of personality and sense of coherence in patients with type-2 diabetes: examining the mediating role of psychological well-being

**DOI:** 10.3389/fpsyg.2024.1375204

**Published:** 2024-02-02

**Authors:** Frontiers Editorial Office

The journal retracts the 27th November 2023 article cited above.

Following publication, concerns were raised regarding the validity of the data in the article. The authors failed to provide the raw data or a satisfactory explanation during the investigation, which was conducted in accordance with Frontiers' policies. Given the concerns, and the lack of raw data, the editors no longer have confidence in the findings presented; the article is therefore retracted.

This retraction was approved by the Chief Editors of Frontiers in Psychology and the Chief Executive Editor of Frontiers. The authors did not agree to this retraction.

